# Factors associated with psychiatric admission from observation beds in the psychiatric emergency room

**DOI:** 10.3389/fpsyt.2026.1857966

**Published:** 2026-08-12

**Authors:** Shreya Kumar, Ryan Serdenes, Jamie Karasin, Linda Callans, Stephanie Fagbemi, Morgan Lewis, James Graham

**Affiliations:** 1 Lewis Katz School of Medicine at Temple University, Philadelphia, PA, United States; 2 Department of Psychiatry, Lewis Katz School of Medicine at Temple University, Philadelphia, PA, United States; 3 Department of Psychiatry, Brigham and Women’s Hospital, Boston, MA, United States; 4 Department of Psychiatry, Women’s Emotional Wellness Center at Main Line Health, King of Prussia, PA, United States

**Keywords:** inpatient psychiatric care, observation unit care, psychiatric admission, psychiatric emergency, psychiatric emergency care, psychiatric emergency triage, psychiatric observation unit, psychiatric symptoms

## Abstract

**Background:**

Psychiatric emergency services frequently utilize observation units to support disposition decisions when diagnostic uncertainty exists, allowing for extended psychiatric supervision to inform medical decision-making. Yet there is limited data describing predictors of inpatient admission following observation, and understanding which factors are associated with this outcome may improve clinical decision-making and resource utilization.

**Methods:**

We conducted a retrospective chart review of adult patients placed in an observation unit within a high-volume urban psychiatric emergency service between April 1 and June 30, 2019. Demographic, psychiatric, and substance-related variables were extracted from the electronic medical record. The primary outcome was disposition to acute inpatient psychiatric admission versus no psychiatric admission. Univariate analyses were performed, followed by multivariable logistic regression to identify factors independently associated with admission.

**Results:**

Among 269 patients placed in observation, 81 (30.1%) were admitted to an acute inpatient psychiatric unit and 188 (69.9%) were not admitted to an acute psychiatric inpatient unit. In multivariable analysis, factors associated with increased odds of psychiatric admission included delusions (aOR 2.94, 95% CI 1.46-5.94), depression (aOR 2.27, 95% CI 1.17-4.43), involuntary commitment status at presentation by either a civilian (aOR 3.65, 95% CI 1.43-9.37) or physician/law enforcement (aOR 2.07, 95% CI 1.10-3.90), and absence of a breathalyzer test (aOR 3.58, 95% CI 1.56-8.19). A decreased likelihood of psychiatric admission was associated with patients with two or more prior observation discharges (aOR 0.26, 95% CI 0.08-0.84), and self-reported substance use within the prior 72 hours (aOR 0.46, 95% CI 0.25-0.84).

**Conclusions:**

In this psychiatric emergency cohort, delusions, depressive symptoms, involuntary commitment status at presentation, and absence of a completed breathalyzer were associated with increased odds of acute psychiatric admission, while self-reported substance use within the prior 72 hours and two or more prior observation discharges were associated with decreased odds. These findings should be interpreted as hypothesis-generating given the observational design, limited study window, and single-site sample. Validation in larger, multi-site, multi-season cohorts is needed before these associations can inform clinical decision-making or be incorporated into disposition prediction tools.

## Introduction

Psychiatric emergency services (PES) have existed since the 1950s and remain a major source of admissions to the approximately 73, 000 inpatient psychiatric beds available in the United States (1, 2). The Temple University Hospital - Episcopal Campus (TUH-EH) Crisis Response Center (CRC) is a psychiatric emergency service that provides emergency psychiatric evaluations, treatment, and referrals for adult patients experiencing behavioral health crises. The TUH-EH CRC serves a dense urban population in Philadelphia, with over eleven thousand patient encounters recorded in 2019.

One treatment option available in the TUH-EH CRC is placement into observation status when there is uncertainty regarding the appropriate clinical disposition. The observation unit serves patients who require additional assessment time, allowing psychiatrists to provide up to 23 hours of supervised evaluation and care until a disposition decision can be made. This distinction is clinically significant, as presentation during an acute crisis may not fully capture a patient’s baseline functioning or the trajectory of their symptoms. In the context of a strained mental health system, characterized by a growing mismatch between the need for psychiatric care and the availability of both emergency psychiatric services and inpatient bed capacity, observation units serve a critical triage function, helping clinicians determine the most appropriate level of care and optimize the use of limited psychiatric resources (3–7). The observation period is used to monitor behavior, obtain collateral information, and evaluate whether substances may be contributing to the patient’s clinical presentation. At the conclusion of the observation period, a final disposition decision is made using all clinical information obtained during the stay.

Despite a long history, research into psychiatric emergency care remains limited, and there is even less literature examining the use of observation beds within PES. While several studies have investigated factors predicting psychiatric admission from PES more broadly, there is no United States based research specifically exploring predictors of admission from PES observation units (8–10). To our knowledge, only one study from Singapore has examined determinants of psychiatric admission from observation units (11). That study identified patient age, past psychiatric history, and psychosocial functioning as significant predictors of admission; however, it did not examine symptom level predictors, substance-related variables, or prior observation utilization patterns, nor was it conducted within the context of the United States psychiatric emergency care system.

We sought to identify demographic and clinical factors associated with inpatient psychiatric admission from observation status in the TUH-EH CRC through a retrospective, observational, cross-sectional chart review. Given the widespread use of psychiatric observation beds within emergency psychiatric care in the United States and the paucity of literature examining disposition outcomes from observation, our findings may inform clinical decision making, systems level care organization, and resource utilization. Clinically, identifying patients at higher likelihood of inpatient admission following observation could allow for earlier initiation of bed searches, reduced delays to definitive psychiatric treatment, and more efficient patient flow within psychiatric emergency services. Such insights may also support clinicians in making timely, evidence informed disposition decisions while optimizing use of limited inpatient and emergency psychiatry resources.

Prior studies have identified psychiatric symptom severity, involuntary commitment status, substance use, and agitation as clinically relevant factors associated with psychiatric admission from emergency settings (9, 10, 12, 13). Building on this literature, we hypothesized that variables related to substance use, agitation, commitment status, and psychiatric symptoms would be associated with psychiatric admission from observation beds in the TUH-EH CRC.

## Methods

Our study is a retrospective Electronic Medical Record (EMR) chart review of patients within the TUH-EH CRC observation unit. The protocol was reviewed and approved by the Temple University Institutional Review Board. All research team members participating in data collection completed Collaborative Institutional Training Initiative (CITI) training. Data was collected, de-identified, and stored in a HIPAA compliant institutional REDCap database. We utilized pre-COVID data over a three-month period, between April 1 and June 30, 2019. For subjects with multiple presentations within the same time period, only their first encounter was utilized within our data set. A three-month study period was selected given the high patient volume of the TUH-EH CRC, which was determined to provide an adequate sample for analysis, and a foundation for potential future larger studies.

A list of variables was developed based on clinical relevance, study objectives, and feasibility of obtaining from chart review. We collected variables related to demographic information, clinical presentation, substance use, psychiatric management, and dispositional outcomes from the current and prior CRC observation periods. Suicidal ideation, homicidal ideation, and involuntary commitment status were analyzed as separate variables. Although suicidal or homicidal ideation may contribute to involuntary commitment decisions, commitment status may also be influenced by other clinical factors including psychosis, inability to care for oneself, or concerns raised through collateral information. Therefore, we considered commitment status to be a distinct variable, as decisions regarding involuntary commitment may be influenced by multiple clinical factors beyond suicidal or homicidal ideation alone. Involuntary commitment status was recorded as present if a formal legal petition filed by a civilian or physician/law enforcement was in place at the time of presentation to the observation unit, irrespective of whether the petition was subsequently upheld or whether the patient was ultimately legally committed. All patients were evaluated by a psychiatrist during their observation period, and clinical symptoms were operationalized from psychiatrist-documented observations at first presentation rather than formal diagnostic criteria or patient self-report. Specifically, psychiatric symptom variables, including depression, delusions, mania, suicidal ideation, and homicidal ideation, reflect clinician-documented findings recorded in the EMR during the psychiatric evaluation and were not derived from structured diagnostic interviews or standardized rating scales. Substance use variables were operationalized as follows: self-reported substance use reflects patient disclosure to the treating clinician as documented in the clinical note; observed intoxication reflects clinician-documented behavioral or physical signs of intoxication at the time of evaluation; urine drug screen results reflect laboratory-confirmed findings from urine toxicology obtained during the encounter; and breathalyzer findings reflect blood alcohol content measured via breathalyzer. Breathalyzer testing was universally attempted for all patients presenting to the observation unit; absence of a recorded result indicates that testing was not completed, and the specific reason for non-completion was not systematically documented in the EMR. We constructed a data dictionary to formalize how chart data is interpreted and extracted by the research team’s data abstractors. All data abstractors attended a training session with the principal investigator (PI). Each chart was reviewed by a single abstractor rather than verified by a second independent reviewer. Additionally, if there was any ambiguous subject specific data, the research team member convened with the PI to ensure accurate data recording.

Our study’s primary outcome was defined as admission to an acute inpatient psychiatric unit versus no psychiatric admission. Patients not admitted to an acute psychiatric inpatient unit were either discharged to the community or referred to substance use treatment. Substance use treatment dispositions refer specifically to non-inpatient-psychiatry and non-dual-diagnosis placements: patients who, following psychiatric evaluation, were determined not to require acute inpatient psychiatric care. We compared subjects admitted to an acute inpatient psychiatric unit with those who were not.

We used SAS version 9.4 (SAS Institute Inc., Cary, NC) for our analyses. Our data analysis included descriptive summary statistics along with univariate analysis. Odds Ratios (OR) along with their respective p-values and confidence intervals were calculated for each variable in the univariate models. For the multivariable analysis, we used stepwise variable selection, which combines forward entry and backward elimination. At each step, forward selection used the Score Chi-Square statistic, entering the not-yet-included variable with the smallest Score Chi-Square p-value below 0.10. Backward elimination used the Wald Chi-Square statistic, removing any included variable whose Wald Chi-Square p-value exceeded 0.05. This iterative forward entry and backward elimination continued until no remaining candidate met the entry criterion and all retained predictors met the stay criterion. Global goodness-of-fit diagnostics and formal multicollinearity assessments were not performed for the multivariable model. The calculated EPV (Events Per Variable) for our final multivariable model was 9. While there is risk of overfitting, our analysis is intended to be exploratory and hypothesis-generating.

## Results

### Study population

Between April 1 and June 30, 2019, there were 269 unique subjects placed in the TUH-EH CRC observation unit. The median age was 40 years (range 18–68). Of these patients, 61.7% were male and 38.3% were female. There were 182 patients (67.7%) identified as Black, 43 (16.0%) as White, and 44 (16.4%) as Hispanic. Of the 269 individuals transferred to the observation unit, 81 (30.1%) were admitted to an acute psychiatric inpatient unit, while 188 (69.9%) were not admitted to an acute psychiatric inpatient unit. Of the 188 patients not admitted to an acute psychiatric inpatient unit, some were discharged to the community while others were referred to substance use treatment; both groups are captured within the no psychiatric admission comparator group. There was no missing variable data for any of the subjects included in the analysis, as confirmed by a systematic review of the REDCap database to verify complete data entry for all included subjects.

### Presenting characteristics

In univariate logistic regression, delusions (OR 2.7, 95% CI 1.46-5.00, p = 0.002), manic symptoms (OR 5.04, 95% CI 1.47-17.25, p = 0.005), and involuntary commitment status at presentation (OR 2.31, 95% CI 1.35-3.93, p = 0.002) were each associated with increased odds of psychiatric admission. In contrast, intoxication was associated with decreased odds of admission (OR 0.39, 95% CI 0.18-0.83; p = 0.011). Of note, suicidal ideation, homicidal ideation, visual hallucinations, and auditory hallucinations were not significantly associated with disposition (p= 0.21, 0.18, 0.50, and 0.94, respectively). Also, the use of intramuscular medication or restraints was not significantly associated with disposition (p=0.11). Notably, manic symptoms (n = 12) demonstrated a wide confidence interval reflecting a small subgroup size and should be interpreted with caution as a potentially unstable estimate.

**Figure 1:**
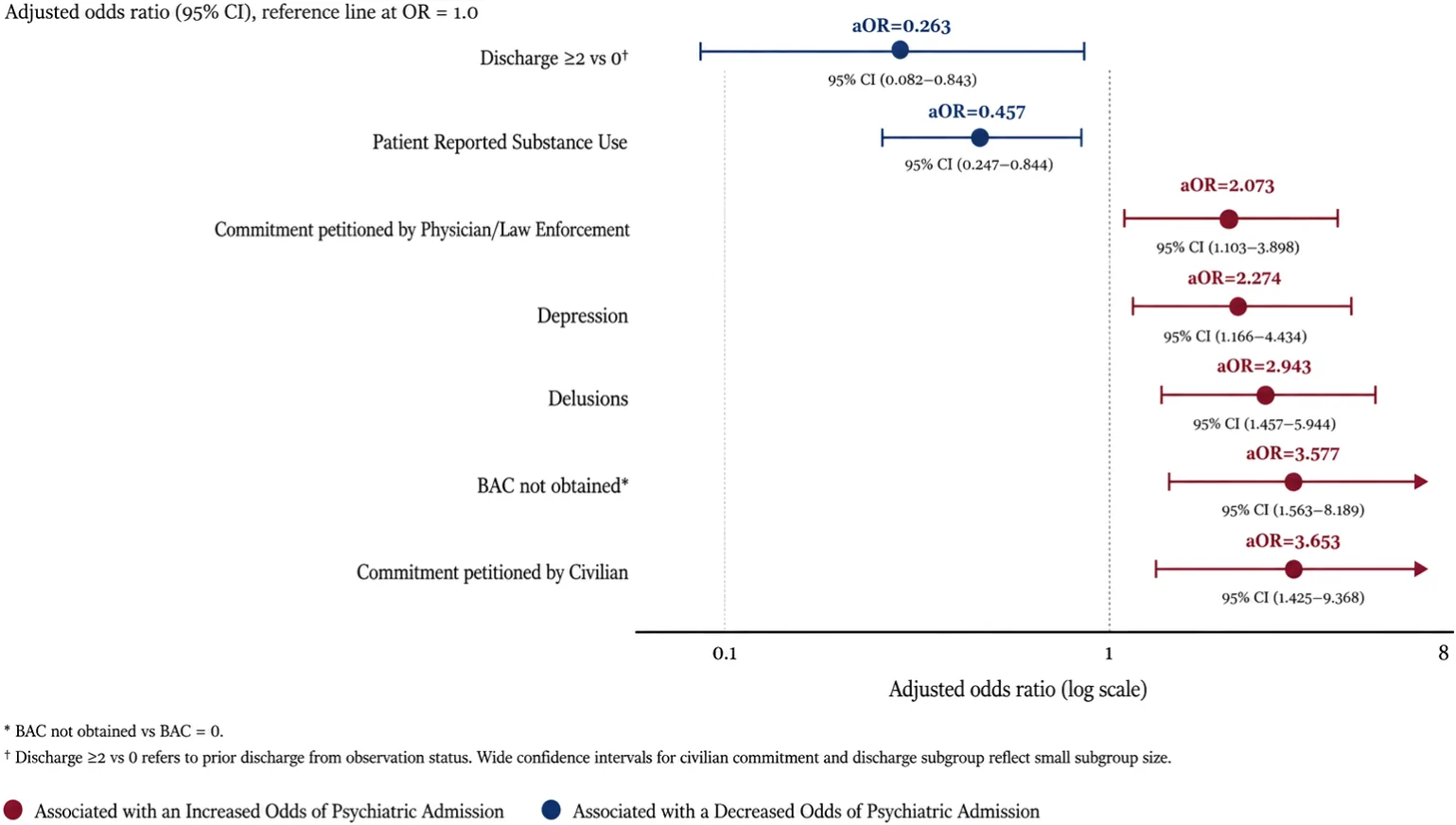
Multivariate analysis results.

In multivariable logistic regression, delusions (aOR 2.94, 95% CI 1.46-5.94) and depression (aOR 2.27, 95% CI 1.17-4.43) were associated with increased odds of acute psychiatric admission, while manic symptoms did not retain statistical significance after adjustment. Involuntary commitment status at presentation by both civilians (aOR 3.65, 95% CI 1.43-9.37) and physician/law enforcement (aOR 2.07, 95% CI 1.10-3.90) remained associated with increased odds of admission. See **Table 1**, and **Figure 1**.

**Table 1:** Multivariate analysis results.

Variable	Adjusted odds ratio	Confidence interval	P-value
Depression	2.274	(1.166, 4.434)	0.0159
Delusions	2.943	(1.457, 5.944)	0.0026
Commitment Petitioned by Civilian	3.653	(1.425, 9.368)	0.0070
Commitment petitioned by Physician/Law Enforcement	2.073	(1.103, 3.898)	0.0236
BAC Not Obtained	3.577	(1.563, 8.189)	0.0026
Patient Reported Substance Use	0.457	(0.247, 0.844)	0.0124
Discharge >2 vs 0	0.263	(0.082, 0.843)	0.0247

### Substance use and alcohol

In univariate analysis, reported substance use (OR 0.43, 95% CI 0.25-0.74, p = 0.002) and history of synthetic cannabinoid use (OR 0.43, 95% CI 0.21-0.91, p = 0.028) were associated with decreased odds of psychiatric admission, whereas absence of a breathalyzer result was associated with increased odds of admission (OR 3.11, 95% CI 1.53-6.31, p = 0.005). In the multivariable model, self-reported substance use within the prior 72 hours remained associated with decreased odds of psychiatric admission (aOR 0.46, 95% CI 0.25-0.84), while absence of a breathalyzer result (BAC) remained associated with increased odds of admission (aOR 3.58, 95% CI 1.56-8.19). See **Table 1**, and **Figure 1**.

### Prior psychiatric history

In univariate analysis, the history of three or more observation presentations in the past year (OR 0.20, 95% CI 0.05-0.89, p = 0.065) and history of two or more prior discharges following observation within the past year (OR 0.27, 95% CI 0.09-0.78, p = 0.007) were associated with lower odds of acute psychiatric admission. Among patients with a history of three or more prior observation stays, 91% did not require acute psychiatric admission. The estimate for three or more observation presentations should be interpreted with caution given the small subgroup size (n = 23). In multivariable analysis, two or more prior observation discharges remained associated with decreased odds of admission (aOR 0.26, 95% CI 0.08-0.84). See **Table 1**, and **Figure 1**.

## Discussion

Despite the widespread use of psychiatric observation units in emergency behavioral health settings, US-based data examining predictors of inpatient psychiatric admission following observation remain limited. This study addresses this gap by identifying clinical factors associated with admission from observation status within a high-volume urban crisis response center. In this cohort, approximately 30% of patients placed in psychiatric observation were ultimately admitted to an acute inpatient unit, while nearly 70% were not admitted to an acute psychiatric inpatient unit. These findings highlight the heterogeneity of clinical trajectories among patients selected for observation-level care and underscore the importance of identifying clinical characteristics that may help inform disposition decisions. More broadly, the ability to observe patients for a limited period may provide valuable time for diagnostic clarification, stabilization, and collateral gathering prior to determining the most appropriate level of care.

Given the retrospective observational design of this study, the associations identified should not be interpreted as causal relationships. With that important limitation in mind, several acute clinical features were associated with increased likelihood of psychiatric admission following observation, with delusions and depressive symptoms emerging as significant psychiatric symptoms after multivariable adjustment. While symptom persistence was not directly measured, these findings suggest that presentations characterized by psychotic or affective symptoms were associated with higher odds of inpatient psychiatric treatment following the observation period.

With respect to symptom level predictors, depressive symptoms emerging in the multivariable model, despite being non-significant in the univariate model, is likely attributable to suppression by co-occurring substance use variables. Given the high prevalence of comorbid substance use and depression in psychiatric populations, univariate analysis may underestimate the association between depressive symptoms and admission by conflating depressive presentations with substance-related ones (14). Manic symptoms demonstrated a large effect size in univariate analysis but did not retain significance after multivariable adjustment, likely reflecting symptom overlap with other clinical domains already captured in the model such as agitation, psychosis, and disorganized behavior, rather than a true absence of clinical effect. The small subgroup size of patients with documented manic symptoms (n = 12) further limits the stability of this estimate and reduces power to detect an association in the multivariable model. The absence of a significant association between suicidal ideation and disposition is also clinically notable. Suicidal ideation is among the most common presenting complaints in psychiatric emergency settings, and its non-significance may reflect the heterogeneity of suicidal ideation as a clinical construct, ranging from passive ideation to active intent, which when treated as a binary variable may lack the granularity needed to meaningfully predict disposition (15). It is also possible that suicidal ideation functions more as a threshold for observation placement than as a differentiator of disposition within the observation population, given that all patients in this cohort had already been selected for extended evaluation.

Turning to commitment status and substance-related predictors, involuntary commitment status at presentation was also associated with higher odds of admission, regardless of whether initiated by a civilian or physician/law enforcement. This likely reflects elevated perceived clinical risk and procedural pathways inherent to involuntary evaluation. In contrast, self-reported substance use was associated with decreased odds of admission. This association may reflect clinical stabilization during observation, diversion to substance use treatment pathways, or other unmeasured factors. Notably, self-report rather than objective urine drug screen findings emerged as significant, which may reflect that the act of disclosing substance use requires insight and communicative capacity, characteristics that themselves signal lower psychiatric acuity and may inform disposition toward non-psychiatric settings (16, 17). Clinically, the observation period serves as a diagnostic tool in this context, allowing time to differentiate substance-induced presentations from primary psychiatric disorders and guiding disposition decisions. Since breathalyzers are universally attempted for every patient in the observation unit, the absence of a recorded BAC indicates that testing was not completed. Based on clinical experience, we suspect this most often reflects patient non-cooperation rather than refusal, clinical omission, or logistical constraints; however, as this was not systematically documented in the EMR, this distinction cannot be confirmed retrospectively. Furthermore, when a BAC can be obtained and accounted for, clinicians may have greater confidence attributing residual symptoms to a primary psychiatric etiology, facilitating more straightforward disposition decisions. Conversely, the inability to obtain this data point may itself signal a presentation too disorganized or agitated to permit standard assessment. Patient non-cooperation with breathalyzer testing may therefore reflect impaired insight or other unmeasured clinical factors associated with higher psychiatric acuity and inpatient admission, though this interpretation should be considered exploratory given the retrospective limitations of this variable (16–19).

Regarding prior observation unit utilization, prior observation unit disposition history was also associated with current disposition outcomes, with patients who had two or more prior observation discharges demonstrating lower odds of acute psychiatric admission during the encounter. This association may reflect recurrent low-acuity utilization patterns. However, alternative explanations, including housing instability, barriers to outpatient care, substance use, help-seeking behavior, and other unmeasured factors, may also contribute to the observed association (20–24). Given that our institution is an urban safety-net hospital serving a high-burden population, these factors likely contribute meaningfully to the repeat utilization patterns observed in our cohort. While help-seeking behavior may contribute to this pattern, this interpretation remains speculative, and alternative explanations, including inadequate outpatient follow-up, housing instability, and habitual low-acuity crisis presentations, warrant equal consideration and underscore the importance of targeting this subgroup for improved care continuity.

The existing literature on predictors of psychiatric admission can be organized into three broad categories: studies examining observation unit cohorts specifically, studies examining general psychiatric emergency service populations, and longitudinal or system-level studies. Importantly, observation unit patients represent a selected subgroup characterized by diagnostic uncertainty or borderline disposition decisions, patients for whom a disposition decision could not be made at initial evaluation. This population differs structurally from general PES cohorts, and comparison should therefore be interpreted with methodological caution through the following synthesis.

Regarding observation unit-specific literature, one prior study has demonstrated that centers with dedicated observation units have lower inpatient admission rates than those without, suggesting that extended evaluation reduces unnecessary hospitalizations (25). To date, only one study, conducted outside the United States in Singapore, has evaluated admission predictors specifically from observation status. That study identified age, prior psychiatric history, and psychosocial functioning as significant factors, and demonstrated that observation was associated with rapid symptom stabilization and reduced self-harming behavior (11). Our study extends this work by identifying symptom-level and substance-related variables associated with admission within an observation population, while also demonstrating an association between prior observation utilization patterns and current disposition, consistent with the Singapore cohort’s findings regarding prior psychiatric history.

Turning to the broader general PES literature, which, as noted above, differs from observation unit cohorts in patient selection and care structure, several findings remain informative for contextualizing our results. It is important to note that many of these studies were conducted outside of the United States, in healthcare systems with different legal frameworks, admission thresholds, and resource constraints, and comparisons should therefore be made cautiously. With respect to symptom-level predictors, international PES studies identified clinical presentations involving delusions as important predictors of inpatient psychiatric admission, a finding similarly observed in our cohort (9, 12). With respect to substance use, a U.S.-based PES study found that alcohol and drug use were associated with a lower probability of admission, consistent with our finding that self-reported substance use was associated with decreased odds of admission (10). A large-scale, five-year retrospective international study has shown that substance use and involuntary hospitalization are strongly associated in psychiatric inpatient populations (26). In our study, however, self-reported substance use was found to be associated with a lower likelihood of hospitalization. This discrepancy may stem from differences in clinical settings, patient groups, and admission thresholds. However, both studies support the clinical significance of substance use in the processes of psychiatric admission and discharge.

With respect to sociodemographic predictors, a prior U.S.-based study found that age, gender, race/ethnicity, homelessness, and employment status were significantly related to hospitalization in an urban PES (10). Our study demonstrates that sociodemographic factors such as gender and race/ethnicity were not associated with disposition after multivariable adjustment, and our primary findings remained robust even after accounting for these variables. The absence of race/ethnicity as a significant variable is notable given the well-documented literature on racial disparities in psychiatric care, including disproportionate rates of involuntary commitment, inpatient admission, and chemical sedation among Black and minority patients (27–29). One possible interpretation is that the structured observation period may promote more equitable, clinically driven disposition decisions by allowing time for symptom evolution, sobriety, and collateral assessment. However, this interpretation is speculative and should be interpreted with caution. The study was not designed to examine disparities, the sample was drawn from a single urban site with a predominantly Black patient population which may limit variability, and unmeasured confounders including provider bias and systemic factors cannot be excluded. Future studies with larger and more demographically diverse samples would be needed to determine whether observation units meaningfully attenuate the influence of sociodemographic factors on psychiatric disposition decisions.

Finally, with respect to system-level factors, it is important to acknowledge that several system-level variables were not captured in this study and represent significant unmeasured contextual influences on disposition. Real-time inpatient psychiatric bed availability, provider-level variability in clinical decision-making, institutional admission thresholds, insurance status, housing status, and availability of substance use or community-based alternatives may have substantially influenced disposition decisions independent of the patient-level clinical variables identified here. These factors are discussed further in the Limitations section, but warrant acknowledgment in the discussion context as well: the associations identified in this study reflect patient-level clinical characteristics and should not be interpreted as fully accounting for the broader systemic and structural determinants of psychiatric emergency disposition.

Taken together, the variables independently associated with inpatient admission in our multivariable analysis begin to sketch a clinical profile that may inform future predictive work. Patients presenting with delusions, depression, or involuntary commitment status at the time of observation-unit entry, and those who do not complete aspects of standard assessment such as the breathalyzer exam, may represent a higher-acuity subgroup more likely to require acute inpatient psychiatric care. Conversely, patients with the preserved communicative capacity to self-report substance use and multiple prior observation-unit discharges without admission may be more amenable to stabilization within the observation setting and subsequent diversion to non-psychiatric pathways. It is important to emphasize that these findings reflect exploratory associations from a single-site retrospective study and should not be interpreted as clinical decision rules. External validation in larger, prospective samples would be necessary before any of these variables could be integrated into formalized disposition algorithms. Nevertheless, these associations may lay the groundwork for future studies examining the potential predictive value of these clinical characteristics in psychiatric emergency disposition.

### Limitations

Our study has several limitations. It was a single-site, retrospective analysis conducted over a limited pre-COVID timeframe, which may limit generalizability. Reliance on electronic medical record documentation introduces the potential for incomplete or inconsistent data capture. Although data abstractors received guidance and had access to the PI for clarification on ambiguous cases, formal inter-rater reliability was not assessed, as each chart was reviewed by a single abstractor rather than verified by multiple team members.

Certain clinical variables, such as the severity or content of psychotic symptoms, were not characterized in detail. Additionally, some patients received medications or restraints prior to arrival in the CRC, which may have influenced clinical presentation and subsequent disposition. Specifically, medications such as antipsychotics and benzodiazepines may produce short-term improvements in psychosis, agitation, anxiety, or behavioral disturbances. The impact of specific treatment interventions during the observation period was not evaluated and warrants investigation in future studies. Post-discharge outcomes were not assessed, including re-presentation to other facilities or later psychiatric admission following discharge from observation.

While breathalyzer testing was universally attempted, the absence of a recorded BAC result could not be further characterized retrospectively, as the specific reason for non-completion, whether patient non-cooperation, refusal, clinical omission, or logistical constraints, was not systematically documented in the EMR; this limits the interpretability of this variable and warrants caution in attributing its association with inpatient admission to any single underlying mechanism.

Several important system-level factors were not captured in this study and represent significant unmeasured confounders. Real-time inpatient psychiatric bed availability, provider-level variability in clinical decision-making, institutional admission thresholds, insurance status, housing status, and the availability of substance use treatment or community-based alternatives may have substantially influenced disposition decisions independent of patient-level clinical variables. The absence of this system-level data limits our ability to fully attribute disposition outcomes to the identified variables and represents an important direction for future research. Additionally, risks associated with not completing goodness-of-fit diagnostics or formal multicollinearity assessments include model instability and overfitting; however, our analysis is intended to be exploratory and hypothesis-generating.

Finally, the three-month single-season study window introduces the possibility of seasonal effects on psychiatric emergency presentations, which are well-documented in the literature (30–32). Patterns of substance use, mood episodes, and crisis presentations may vary across seasons, and the generalizability of findings from a single spring quarter may be limited. Future studies spanning multiple seasons and longer time periods would strengthen the external validity of these findings.

### Future directions

Future studies should include larger, multi-site cohorts and assess post-discharge outcomes to better understand the longer-term impact of observation-based disposition decisions. Comparing pre- and post-COVID observation outcomes may yield insights into evolving patterns of psychiatric emergencies. Further refinement of symptom-level variables and evaluation of dynamic clinical changes during observation may provide additional insight into disposition outcomes. These findings may ultimately inform the development of clinical decision support tools to assist clinicians in identifying patients more likely to undergo inpatient psychiatric admission while preserving the benefits of observation for appropriate patients.

## Conclusion

In this single-site, retrospective cohort of psychiatric emergency patients managed in an observation setting, delusions, depressive symptoms, and involuntary commitment status at presentation were statistically associated with increased odds of acute inpatient psychiatric admission, while self-reported substance use within the prior 72 hours and two or more prior observation discharges were associated with decreased odds. These findings are preliminary and should not be interpreted as causal given the observational design, single-site sample, and limited single-season study window. Future studies using larger, multi-site cohorts spanning multiple seasons are needed to validate these associations and determine whether they can be incorporated into clinical decision-support tools to assist with disposition risk stratification and optimization of psychiatric emergency workflow and resource utilization.

## Data Availability

The data analyzed in this study is subject to the following licenses/restrictions: The dataset contains protected patient information and is restricted due to privacy regulations (HIPAA). Access is limited to authorized researchers within the institution, and it cannot be shared publicly. However, aggregated results are presented in the article. Requests to access these datasets should be directed to JK, Jamie.Karasin@tuhs.temple.edu.
