# Supplementary material for: Factors associated with psychiatric admission from observation beds in the psychiatric emergency room
Source: Front Psychiatry. 2026 Aug 12;17:1857966. doi: 10.3389/fpsyt.2026.1857966 (PMC13506952; doi:10.3389/fpsyt.2026.1857966)
Supplement: Supplementary file 1 [file SupplementaryFile1.docx]

**Appendix:**

Supplementary Table 1: Presenting Psychiatric Symptoms

|  |  | Disposition | |  |  |
| --- | --- | --- | --- | --- | --- |
| Initial Symptoms | Overall  (N=269)  N (%) | Acute Psychiatric Inpatient (N=81)  N (%) | No Psychiatric Admission (N=188)  N (%) | Odds Ratio | P value |
| Suicidal Ideation | 138 (51.3%) | 37 (26.8%) | 101 (73.2%) | 0.72  (0.43, 1.22) | 0.21 |
| Depression | 97 (36.1%) | 30 (30.9%) | 67 (69.1%) | 1.06 (0.62, 1.82) | 0.84 |
| Auditory hallucinations | 83 (30.9%) | 25 (30.1%) | 58 (69.9%) | 1.00 (0.57, 1.76) | 0.94 |
| Disorganized behavior | 67 (24.9%) | 26 (38.8%) | 41 (61.2%) | 1.69 (0.95, 3.03) | 0.078 |
| Intoxication | 55 (20.4%) | 9 (16.4%) | 46 (83.6%) | 0.39 (0.18, 0.83) | 0.011 |
| Delusions | 54 (20.1%) | 26 (48.1%) | 28 (51.9%) | 2.70 (1.46, 5.00) | 0.002 |
| Aggression | 46 (17.1%) | 16 (34.8%) | 30 (65.2%) | 1.30 (0.66, 2.54) | 0.43 |
| Homicidal Ideation | 44 (16.4%) | 17 (38.6%) | 27 (61.4%) | 1.58 (0.81, 3.10) | 0.18 |
| Visual Hallucinations | 17 (6.3%) | 6 (35.3%) | 11 (64.7%) | 1.68(95%CI: 0.63–4.47) | 0.50 |
| Mania | 12 (4.5%) | 8 (66.7%) | 4 (33.3%) | 5.04 (1.47, 17.25) | 0.005 |
| Failure to care for self | 9 (3.3%) | 5 (55.6%) | 4 (44.4%) | 3.03 (0.79, 11.58) | 0.098 |
| Commitment, N%) - No | 145 (53.9%) | 32 (22.1%) | 113 (77.9%) | Reference | 0.002 |
| Commitment, N%) - Yes | 124 (46.1%) | 49 (39.5%) | 75 (60.5%) | 2.31 (1.35, 3.93) | 0.002 |
| Commitment, N%) - No | 145 (53.9%) | 32 (22.1%) | 113 (77.9%) | Reference | 0.003 |
| Commitment, N%) - Yes by civilian | 28 (10.4%) | 14 (50.0%) | 14 (50.0%) | 3.53 (1.53, 8.17) | 0.003 |
| Commitment, N%) - Yes by physician/law enforcement | 96 (35.7%) | 35 (36.5%) | 61 (63.5%) | 2.03 (1.14, 3.59) | 0.003 |

Supplementary Table 2: Demographics

|  |  | Disposition | |  |  |
| --- | --- | --- | --- | --- | --- |
|  | Overall  (N=269)  N (%) | Acute Psychiatric Inpatient (N=81)  N (%) | No Psychiatric Admission (N=188)  N (%) | Odds Ratio | P value |
| Age - Mean (SD) | 40.6 (10.8) | 41.1 (11.0) | 40.4 (10.7) | 1.06 (0.83, 1.35) | 0.63 |
| Gender, N (%)  Female | 103 (38.3%) | 38 (36.9%) | 65 (63.1%) | 1.67 (0.98, 2.84) | 0.066 |
| Gender, N (%)  Male | 166 (61.7%) | 43 (25.9%) | 123 (74.1%) | Reference | 0.066 |
| Race/Ethnicity, N (%) - Black | 182 (67.7%) | 57 (31.3%) | 125 (68.7%) | 1.72 (0.78, 3.83) | 0.34 |
| Race/Ethnicity, N - Hispanic | 44 (16.4%) | 15 (34.1%) | 29 (65.9%) | 1.95 (0.75, 5.12) | 0.34 |
| Race/Ethnicity, N (%) - White | 43 (16.0%) | 9 (20.9%) | 34 (79.1%) | Reference | 0.34 |

Supplementary Table 3: Substance Use

|  |  | Disposition | |  |  |
| --- | --- | --- | --- | --- | --- |
|  | Overall  (N=269)  N (%) | Acute Psychiatric Inpatient (N=81)  N (%) | No Psychiatric Admission (N=188)  N (%) | Odds Ratio | P value |
| BAC, N | 231 | 61 | 170 | 0.50 (0.14, 1.81) | 0.29 |
| BAC2, N (%) 0 | 209 (77.7%) | 55 (26.3%) | 154 (73.7%) | Reference | 0.005 |
| BAC2, N (%) >0 | 22 (8.2%) | 6 (27.3%) | 16 (72.7%) | 1.05 (0.39, 2.82) | 0.005 |
| BAC2, N (%) n/a | 38 (14.1%) | 20 (52.6%) | 18 (47.4%) | 3.11 (1.53, 6.31) | 0.005 |
| Amphetamines, N (%) | 16 (5.9%) | 5 (31.3%) | 11 (68.8%) | 1.06 (0.36, 3.15) | 0.89 |
| Barbiturates, N (%) | 7 (2.6%) | 1 (14.3%) | 6 (85.7%) | 0.38 (0.04, 3.20) | 0.56 |
| BZD, N (%) | 57 (21.2%) | 20 (35.1%) | 37 (64.9%) | 1.34 (0.72, 2.49) | 0.37 |
| Cocaine, N (%) | 102 (37.9%) | 29 (28.4%) | 73 (71.6%) | 0.88 (0.51, 1.51) | 0.63 |
| Opiates, N (%) | 29 (10.8%) | 5 (17.2%) | 24 (82.8%) | 0.45 (0.17, 1.22) | 0.11 |
| Oxycodone, N (%) | 4 (1.5%) | 1 (25.0%) | 3 (75.0%) | N/a | 0.79 |
| Marijuana, N (%) | 87 (32.3%) | 20 (23.0%) | 67 (77.0%) | 0.59 (0.33, 1.06) | 0.077 |
| PCP, N (%) | 48 (17.8%) | 12 (25.0%) | 36 (75.0%) | 0.73 (0.36, 1.50) | 0.44 |
| UDS Negative, N (%) | 37 (13.8%) | 14 (37.8%) | 23 (62.2%) | 1.50 (0.73, 3.09) | 0.29 |
| UDS Not performed, N (%) | 41 (15.2%) | 12 (29.3%) | 29 (70.7%) | 0.95 (0.46, 1.98) | 0.93 |
| Reported Substance use, N (%) - No | 84 (31.2%) | 36 (42.9%) | 48 (57.1%) | Reference | 0.002 |
| Reported Substance use, N (%) - Yes | 185 (68.8%) | 45 (24.3%) | 140 (75.7%) | 0.43 (0.25, 0.74) | 0.002 |
| Synthetic cannabinoid, N (%) - No | 213 (79.2%) | 71 (33.3%) | 142 (66.7%) | Reference | 0.028 |
| Synthetic cannabinoid, N (%) - Yes in any capacity | 56 (20.8%) | 10 (17.9%) | 46 (82.1%) | 0.43 (0.21, 0.91) | 0.028 |
| Synthetic cannabinoid, N (%) - No | 213 (79.2%) | 71 (33.3%) | 142 (66.7%) | Reference | 0.032 |
| Synthetic cannabinoid, N (%) - Yes during previous encounters but not this one | 21 (7.8%) | 6 (28.6%) | 15 (71.4%) | 0.80 (0.30, 2.15) | 0.032 |
| Synthetic cannabinoid, N (%) - Yes during this presentation | 35 (13.0%) | 4 (11.4%) | 31 (88.6%) | 0.26 (0.09, 0.76) | 0.032 |
| Buprenorphine, N (%) | 10 (3.7%) | 2 (20.0%) | 8 (80.0%) | N/a | 0.61 |
| Buprenorphine Unavailable, N (%) | 22 (8.2%) | 3 (13.6%) | 19 (86.4%) | N/a | 0.071 |
| Methadone, N (%) | 10 (3.7%) | 2 (20.0%) | 8 (80.0%) | N/a | 0.61 |
| Reported Opioid Use | 235 (87.4%) | 70 (29.8%) | 165 (70.2%) | N/a | 0.77 |

Supplementary Table 4. Prior Psychiatric History

|  |  | Disposition | |  |  |
| --- | --- | --- | --- | --- | --- |
|  | Overall  (N=269)  N (%) | Acute Psychiatric Inpatient (N=81)  N (%) | No Psychiatric Admission (N=188)  N (%) | Odds Ratio | P value |
| # CRC Encounters (12 months), N | 269 | 81 | 188 | 0.82 (0.67, 1.01) | 0.065 |
| # CRC Encounters (12 months), N - Mean (SD) | 2.7 (6.8) | 1.5 (1.9) | 3.2 (8.0) | 0.82 (0.67, 1.01) | 0.065 |
| # Previous CRC Encounters, N (%): 0 | 111 (41.3%) | 34 (30.6%) | 77 (69.4%) | Reference | 0.30 |
| # Previous CRC Encounters, N (%): 1-2 | 86 (32.0%) | 30 (34.9%) | 56 (65.1%) | 1.21 (0.67, 2.21) | 0.30 |
| # Previous CRC Encounters, N (%): >=3 | 72 (26.8%) | 17 (23.6%) | 55 (76.4%) | 0.70 (0.36, 1.38) | 0.30 |
| # Previous 23 hr obs (12 months), N | 269 | 81 | 188 | 0.76 (0.59, 0.99) | 0.039 |
| # Previous 23 hr obs (12 months), N - Mean (SD) | 0.8 (1.9) | 0.4 (0.8) | 0.9 (2.2) | 0.76 (0.59, 0.99) | 0.039 |
| # Previous 23 hr obs, N (%): 0 | 184 (68.4%) | 59 (32.1%) | 125 (67.9%) | Reference | 0.065 |
| # Previous 23 hr obs, N (%): 1-2 | 62 (23.0%) | 20 (32.3%) | 42 (67.7%) | 1.01 (0.54, 1.87) | 0.065 |
| # Previous 23 hr obs, N (%): >=3 | 23 (8.6%) | 2 (8.7%) | 21 (91.3%) | 0.20 (0.05, 0.89) | 0.065 |
| # Previous 23s to admission, N - Mean (SD) | 0.2 (0.4) | 0.1 (0.3) | 0.2 (0.4) | 0.96 (0.89, 1.03) | 0.27 |
| # Previous 23s to admission, N (%): 0 | 232 (86.2%) | 72 (31.0%) | 160 (69.0%) | Reference | 0.39 |
| # Previous 23s to admission, N (%): >=1 | 37 (13.8%) | 9 (24.3%) | 28 (75.7%) | 0.71 (0.32, 1.59) | 0.39 |
| # Previous 23s to discharge, N | 269 | 81 | 188 | 0.75 (0.56, 1.01) | 0.060 |
| # Previous 23s to discharge, N - Mean (SD) | 0.6 (1.8) | 0.3 (0.7) | 0.8 (2.1) | 0.75 (0.56, 1.01) | 0.060 |
| # Previous 23s to discharge, N (%): 0 | 198 (73.6%) | 62 (31.3%) | 136 (68.7%) | Reference | 0.007 |
| # Previous 23s to discharge, N (%): 1 | 34 (12.6%) | 15 (44.1%) | 19 (55.9%) | 1.73 (0.83, 3.63) | 0.007 |
| # Previous 23s to discharge, N (%): >=2 | 37 (13.8%) | 4 (10.8%) | 33 (89.2%) | 0.27 (0.09, 0.78) | 0.007 |
| # Acute admissions, N - Mean (SD) | 269 | 81 | 188 | 0.97 (0.71, 1.32) | 0.85 |
| # Acute admissions, N (%): 0 | 198 (73.6%) | 56 (28.3%) | 142 (71.7%) | Reference | 0.26 |
| # Acute admissions, N (%): >=1 | 71 (26.4%) | 25 (35.2%) | 46 (64.8%) | 1.38 (0.77, 2.45) | 0.26 |

Supplementary Table 5. Management Details

|  |  | Disposition | |  |  |
| --- | --- | --- | --- | --- | --- |
|  | Overall  (N=269)  N (%) | Acute Psychiatric Inpatient (N=81)  N (%) | No Psychiatric Admission (N=188)  N (%) | Odds Ratio | P value |
| Agitation Intervention, N (%) - No | 219 (81.4%) | 61 (27.9%) | 158 (72.1%) | Reference | 0.11 |
| Agitation Intervention, N (%) - Yes | 50 (18.6%) | 20 (40.0%) | 30 (60.0%) | 1.73 (0.91, 3.27) | 0.11 |
| Agitation Intervention, N (%) - Both IM meds and restraints | 14 (5.2%) | 5 (35.7%) | 9 (64.3%) | 1.44 (0.46, 4.47) | 0.22 |
| Agitation Intervention, N (%) - IM meds | 36 (13.4%) | 15 (41.7%) | 21 (58.3%) | 1.85 (0.90, 3.82) | 0.22 |
| Agitation Intervention, N (%) - None | 219 (81.4%) | 61 (27.9%) | 158 (72.1%) | Reference | 0.22 |
